# Optimizing multi-omics data imputation with NMF and GAN synergy

**DOI:** 10.1093/bioinformatics/btae674

**Published:** 2024-11-15

**Authors:** Md Istiaq Ansari, Khandakar Tanvir Ahmed, Wei Zhang

**Affiliations:** Department of Computer Science, University of Central Florida, Orlando, FL 32816, United States; Department of Genomics and Bioinformatics Cluster, University of Central Florida, Orlando, FL 32816, United States; Department of Computer Science, University of Central Florida, Orlando, FL 32816, United States; Department of Genomics and Bioinformatics Cluster, University of Central Florida, Orlando, FL 32816, United States; Department of Computer Science, University of Central Florida, Orlando, FL 32816, United States; Department of Genomics and Bioinformatics Cluster, University of Central Florida, Orlando, FL 32816, United States

## Abstract

**Motivation:**

Integrating multiple omics datasets can significantly advance our understanding of disease mechanisms, physiology, and treatment responses. However, a major challenge in multi-omics studies is the disparity in sample sizes across different datasets, which can introduce bias and reduce statistical power. To address this issue, we propose a novel framework, OmicsNMF, designed to impute missing omics data and enhance disease phenotype prediction. OmicsNMF integrates Generative Adversarial Networks (GANs) with Non-Negative Matrix Factorization (NMF). NMF is a well-established method for uncovering underlying patterns in omics data, while GANs enhance the imputation process by generating realistic data samples. This synergy aims to more effectively address sample size disparity, thereby improving data integration and prediction accuracy.

**Results:**

For evaluation, we focused on predicting breast cancer subtypes using the imputed data generated by our proposed framework, OmicsNMF. Our results indicate that OmicsNMF consistently outperforms baseline methods. We further assessed the quality of the imputed data through survival analysis, revealing that the imputed omics profiles provide significant prognostic power for both overall survival and disease-free status. Overall, OmicsNMF effectively leverages GANs and NMF to impute missing samples while preserving key biological features. This approach shows potential for advancing precision oncology by improving data integration and analysis.

**Availability and implementation:**

Source code is available at: https://github.com/compbiolabucf/OmicsNMF.

## 1 Introduction

The biological sciences have seen a significant transition in recent years, characterized by both a significant drop in costs and a quick improvement in high-throughput omics technologies. This change has resulted in an unprecedented rise in the production of high-throughput biological data, offering scientists a comprehensive and detailed understanding of biological processes. One significant development arising from this technological surge is the advent of multi-omics research. This paradigm involves the simultaneous acquisition of various omics data types, such as genomics, epigenomics, transcriptomics, and proteomics, from the same set of biological samples (Gomez-Cabrero *et al.* 2014). Analysis of these omics data can help bring revolutionary advancement in many aspects especially in biomedical research. The interconnected nature of these omics data types through complex networks makes their integration a challenging task (Hawe *et al.* 2019). It is well-established that integrating multiple omics data provides superior insights compared to analyzing single omics data alone (Subramanian *et al.* 2020, Wörheide *et al.* 2021, Ahmed *et al.* 2023b). There have been a lot of research in finding the proper way to integrate multi-omics datatypes. However, the biggest obstruction in the way forward seems to be handling missing data in different omics profiles.

Missing value imputation studies represents a pivotal domain within multi-omics research addressing different types of missing data problems (Ahmed *et al.* 2023a). Several imputation methods are available for the scenario where only a portion of a sample is missing. Traditional statistical and machine learning based algorithms like regression (Tibshirani 1996, Seber and Lee 2012) and k-nearest neighbor ensemble based techniques (Troyanskaya *et al.* 2001, Lee and Styczynski 2018) have been proposed to solve the imputation problem. Recently, deep learning-based models have also emerged as effective solutions for missing data imputation. Generative Adversarial Networks (GANs) (Goodfellow *et al.* 2014) are prominent in the deep learning-based imputation models because of its exceptional ability to generate reliable synthetic data. Initially applied for image completion tasks (Tran *et al.* 2017), GANs are now being used across various domains to solve diverse problems (Isola *et al.* 2017, Zhang *et al.* 2017, Ahmed *et al.* 2021). Generative Adversarial Imputation Nets (GAIN) (Yoon *et al.* 2018), for example, introduced a novel approach by using GANs for data imputation, incorporating a conditional input mask to differentiate observed data from missing data in each sample. However, imputing a completely missing modality for a sample poses a unique imputation challenge. In omics domain, one omics profile may be collected for a patient, while another may be missing due to factors like limited time, expensive data collection procedures, or patient non-attendance during data collection. In this scenario the completely missing modality (target modality) can be imputed from another modality (source modality) collected from the same set of samples. To address this, several studies have focused on imputing completely missing omics profiles. Classical methods such as k-nearest neighbor (Dong *et al.* 2019) and multi-factor analysis (MFA) (Voillet *et al.* 2016) have been proposed to solve this problem. These methods often assume linear relationships between different omics profiles and samples, limiting their ability to capture the non-linear interactions inherent in genomic data. Recent advancements in omics data imputation include machine learning approaches like TDimpute (Zhou *et al.* 2020) and OmiTrans (Zhang and Guo 2022). TDimpute utilizes a fully connected network optimized with mean squared error (MSE) loss while OmiTrans employs a GAN-based model combining MSE and adversarial loss for missing sample generation. Although both methods theoretically model the non-linear relationships in omics data, they are still focused on minimizing the mean square error between the available target samples and the generated samples from the model which creates a bias towards available datasets specially when the sample size is not large enough to fit a neural network.

In this work, we propose a GAN-based framework with a loss function that incorporates non-negative matrix factorization (NMF) (Lee and Seung 1999). NMF is an efficient technique for reducing high-dimensional omics data to a low-dimensional structure, effectively capturing complex relationships between multiple omics profiles (Stein-O’Brien *et al.* 2018). This algorithm has been employed not only to identify complex heterogeneous networks in multi-modal omics data (Yang and Michailidis 2016) but also to impute missing values (Xu *et al.* 2021). Typically, when the target modality has missing samples compared to the source modality, these missing samples are not used in training due to the lack of ground truth in the target modality and are instead used for inference in a trained model. However, by including NMF loss in our framework, we can incorporate these missing subsets even during training, providing a significant advantage for GAN training. While NMF alone, as a statistical method, cannot fully address the limitations of complete missing value imputation, combining it with the generative power of GANs offers a robust solution. We have designed a framework named OmicsNMF to achieve complete missing imputation of target omics profiles from source omics profiles.

The remainder of this article is organized as follows. Section 2.1 provides a high-level description of the framework, followed by the technical details in Section 2.2. We then discuss the baseline methods used for comparison in Section 2.3 and present the dataset, experimental setup, and results in Section 3. Finally, we address the limitations of our work and propose future directions in Sections 4 and 5.

## 2 Materials and methods

In this section, we describe the proposed method, OmicsNMF, and the mathematical notations used in this study for multi-omics imputation. We first present an overview of the framework and then provide a detailed methodology.

### 2.1 Overview of the framework

**Figure 1.**
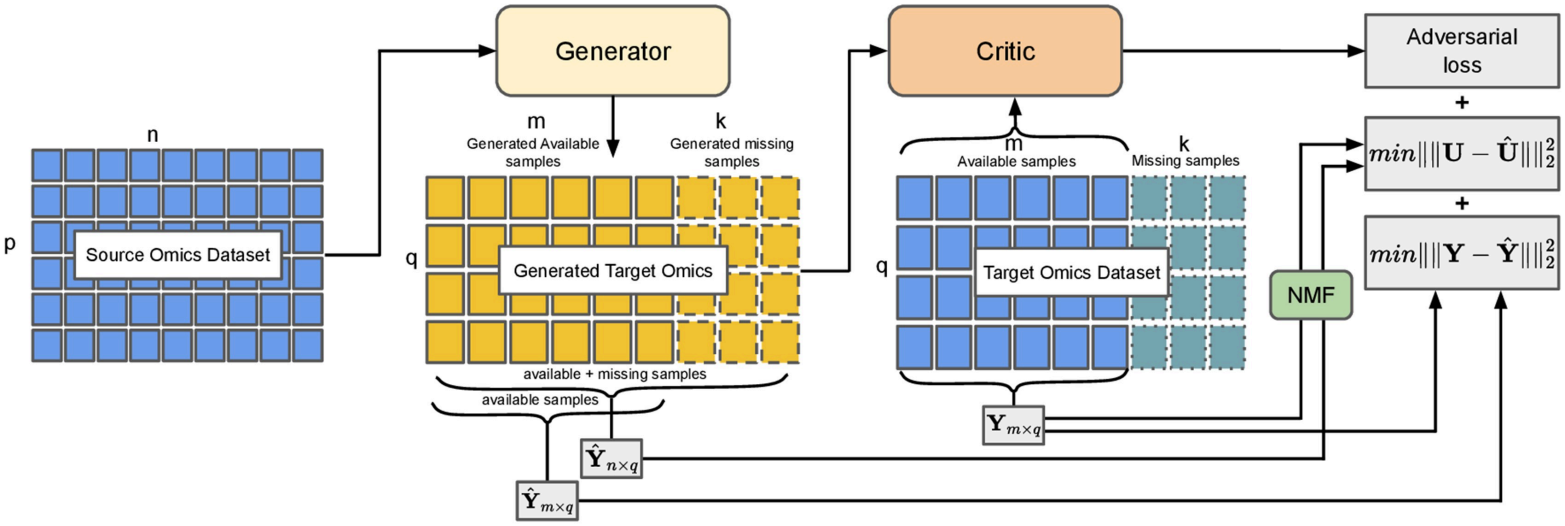
The overview of the OmicsNMF framework. The framework aims to train a Generator to synthesize target omics data from source omics data and a Critic to differentiate between real and synthetic samples through adversarial training. The Critic is trained using adversarial loss, while the Generator’s training is guided by minimizing both the NMF loss between synthetic and real target omics profiles and the MSE loss.

Missing sample imputation facilitates comprehensive analyses and enhances biological insights in multi-omics datasets. In this study, we focus on the imputation of completely missing samples from one omics modality using observed samples from multi-omics profiles so that the entire multi-omics dataset can be utilized for downstream tasks. The overall proposed framework is illustrated in Fig. 1. OmicsNMF is based on a GAN, which uses the source omics profile as input instead of random noise to generate the target omics profile. GANs have gained substantial attention in recent years due to their proficiency in generating realistic synthetic data, making them an ideal foundation for this framework. Typically, GAN frameworks consist of two key components: a generator and a discriminator. The generator generates synthetic data samples that mimic the distribution of the target dataset, while the discriminator learns to distinguish between real and synthetic samples. Through an iterative training process, the generator strives to produce synthetic samples that are indistinguishable from real ones.

However, conventional GAN models often struggle to preserve sample-specific information when generating samples. To address this challenge, our framework focuses on imputing missing samples while maintaining these specific characteristics. Instead of using random noise, we use another omics profile from the same sample as input, which encourages the imputed values to retain sample-specific features. By leveraging the neural networks within the GAN framework, our method more effectively models the complex relationships between omics profiles.

Unlike traditional GANs, which rely solely on adversarial loss, we propose an additional loss based on NMF combined with MSE loss for training. NMF decomposes the data into two matrices: one representing the cluster memberships of each sample, and the other representing the centroids of these clusters. For omics data, it is crucial that the cluster representation remains consistent across different subsets of available samples, and the centroids of missing samples should closely resemble those calculated from the available samples of the target omics. To achieve this, we calculate the cluster centroids for each mini-batch and compare them to the pre-calculated centroids derived from the available samples. This enables us to compute the loss for missing samples without requiring ground truth values, unlike traditional MSE loss. By incorporating missing samples in this manner, our approach enhances the modeling of the translation from source omics to target omics and enables the GAN to learn more robust feature representations. While NMF may have limitations in handling non-linearity, combining it with GAN helps overcome these challenges. Additionally, since NMF’s performance can be sensitive to initial conditions, we set a high maximum iteration count to ensure proper convergence. The MSE loss ensures that the generated dataset aligns with the original target omics profile. Finally, we evaluate OmicsNMF through phenotype prediction using the generated data, which includes the previously missing samples. A summary of the notations used in this study is provided in Table 1.

**Table 1.** Notations.

Name	Definition
*p*, *q*	number of features for source omics and target omics profiles, respectively
$X\in R^{n\times p}$	source omics profile and *n* is the number of available samples
$Y\in R^{m\times q}$	target omics profile and *m* is the number of available samples
$\hat{Y}\in R^{n\times q}$	Generated target omics expression including *k* missing samples, where k=n−m
*G*	Generator
*C*	Critic

### 2.2 Network architecture

Our proposed framework uses the Wasserstein Generative Adversarial Network (wGAN) (Arjovsky *et al.* 2017), a modified version of GAN known for its efficient training capabilities and ability to overcome common GAN training problems such as mode collapse and vanishing gradients. The wGAN architecture consists of two neural networks: the generator (*G*) and the discriminator, referred to as the critic (*C*). These networks engage in an adversarial training process to iteratively refine the generated samples. The generator aims to produce data that is indistinguishable from the real samples, while the critic tries to differentiate between real and synthetic data. GANs are typically implemented with a decoder fashioned generator (Wu *et al.* 2016) and a binary classifier discriminator model. However, as we input source omics to the generator instead of random noise vector and generate the target omics, our generator is designed using an encoder-decoder architecture (Cho *et al.* 2014). Given that our domain of interest is a 1D-signal domain, we implement a shallow fully-connected encoder-decoder network as the generator and a shallow fully-connected model with a single output node as the critic. The generator is a fully connected neural network with two hidden layers consisting of 512 and 768 nodes, respectively. The critic also has two hidden layers with 256 and 128 nodes, respectively, and has a single node in the output layer. All layers in the generator and the critic are followed by ReLU (rectified linear unit) activation function. The critic is expected to assign higher scores to real samples than to synthetic ones. The generator of the framework is designed to generate missing samples of target omics, ***Y***, from source omics, ***X***. Therefore, the input to the generator is the source omics ***X***, as shown in Equation (1) below:

(1) $${\hat{Y}}_{n\times q}=G(X_{n\times p}).$$

As described in Table 1, *n* represents the number of samples in the source omics, and *m* represents the number of samples in the target omics, where k=n−m and *k* is the number of missing samples. Additionally, *p* and *q* are the number of features in the source and target omics, respectively. The objective function for training the critic is given by Equation (2), where ***Y*** represents the true target omics profile and $\hat{Y}$ represents the generated target omics profile. The loss function aims to train the critic to distinguish between real and synthetic samples.

(2) $$L_{C}=C({\hat{Y}}_{m\times q})-C(Y_{m\times q}).$$

NMF decomposes a non-negative data matrix into two non-negative matrices, typically representing basis vectors and coefficients, such that their product approximates the original data matrix. We use this factorization to decompose the available target omics (***Y***) and generated omics ($\hat{Y}$) into two matrices, ***U*** and ***V***, as shown in Equations (3) and (4) below:

(3) $$Y_{m\times q}=V_{m\times c}\times U_{c\times q},$$

(4) $${\hat{Y}}_{n\times q}={\hat{V}}_{n\times c}\times {\hat{U}}_{c\times q},$$

where *c* represents the number of clusters, $V_{m\times c}$ represents the cluster membership of each sample, and $U_{c\times q}$ gives the values of the centroids of those clusters for ***Y***. We perform the same decomposition on $\hat{Y}$ to obtain the centroid matrix ${\hat{U}}_{c\times q}$. Note that ${\hat{U}}_{c\times q}$, which comes from the generated target omics, includes the *k* samples that are missing from the target omics data but are available in the source omics data. In Fig. 1, the NMF block calculates both ***U*** and ***V***, and we use only the centroid matrix ***U*** to calculate the NMF loss. This decomposition is performed for each mini-batch, using the generator’s output with random initialization and a large maximum iteration count to ensure convergence. The objective function is designed to minimize the distance between the centroid matrix derived from the available target omics and that from the generated target omics. It is to ensure that the properties of *q* features in $\hat{Y}$ after imputation of *k* samples are similar to ***Y*** which only contains true values.

(5) $$\begin{array}{l} L_{G}=C(G(X_{n\times p}))-\alpha (||U_{c\times q}-{\hat{U}}_{c\times q}|{|}_{2}^{2})\\ -\beta (||Y_{m\times q}-{\hat{Y}}_{m\times q}|{|}_{2}^{2}). \end{array}$$

Equation (5) represents the overall objective function for the generator. The first term is the critic loss, which aims to fool the critic into identifying the generated samples as real ones. This forces the generator to learn to produce samples that imitates the distribution of real samples and are hard to distinguish. The second term ensures that the cluster representation of the original target omics and the generated target omics are similar by minimizing the distance between them. This term also allows us to incorporate the completely missing samples into the loss function, as the centroid matrix represents an overall distribution of the omics profile rather than specific samples. From Equations (3) and (4), it is evident that it is not necessary to have the same number of samples to generate ***U*** for both the available target omics and the generated target omics. Therefore, we can calculate $\hat{U}$ including the missing samples and thus calculate the NMF loss. Finally, the third term represents the *L*_2_ norm calculated between the true target omics and the generated target omics profile (MSE loss). This part is calculated only over the available samples in the target omics data. The two tuning parameters *α* and *β* control the weight put on the last two terms.

Finally, after training OmicsNMF, we use the generator to impute missing samples for the target omics using the available target and source omics profiles. Further testing and evaluations are then performed on the generated omics profile.

### 2.3 Baselines and evaluation methods

For performance comparison, several previous methods were implemented as baselines using the same data splitting setup to ensure the results are comparable with OmicsNMF. OmiTrans (Zhang and Guo 2022) is the latest proposed method for imputing one missing omics profile from another available profile, using a vanilla GAN architecture combined with MSE loss during the generator training step. TDimpute (Zhou *et al.* 2020) is another method that uses MSE loss with a simple feed-forward network. A statistical machine learning method, TOBMI (Dong *et al.* 2019), is also implemented. TOBMI uses the k-nearest neighbor algorithm to find the nearest neighbors from *m* available samples in source omics data and averages those samples in the target omics data to impute the missing samples. Additionally, we compare our method with traditional linear regression. Finally, we also evaluate a modified version of our framework where the MSE loss term is removed and only the NMF loss is used for training the GAN model. This version is referred to as “NMF Only” in the tables and figures.

One of the main reasons for investigating the imputation of omics profiles is to address completely missing samples for downstream tasks and analyses. First, we evaluate the quality of the imputed omics profiles for the phenotype classification task. For this evaluation, we use a basic Random Forest classifier to predict phenotypes using the generated omics profiles, including the imputed missing samples. Second, we perform survival analysis on the imputed omics profiles to assess the quality of the imputed data. We use an Elastic Net-penalized Cox proportional hazards model (Simon *et al.* 2011) to analyze the relationship between patients’ overall survival time or disease-free time and their omics profiles. The Elastic Net penalty combines *L*_1_-norm and *L*_2_-norm penalties by maximizing the following log-likelihood function:

(6) $$\text{log}\;L(\beta )-\alpha \left(r\sum_{i=1}^{q}|\beta_{i}|+\frac{1-r}{2}\sum_{i=1}^{q}\beta_{i}^{2}\right),$$

where $\beta \in R^{1\times q}$ is the vector of regression coefficients or risk coefficients, L(β) is the partial likelihood of the Cox model, α≥0 is a hyperparameter that controls the amount of shrinkage, r∈[0,1] is the relative weight of the *L*_1_-norm and *L*_2_-norm penalties, and *β_i_*, where i∈[1,q], represents the coefficient for the *i*th genomic feature in the omics data. We split the data into training and test sets with an 80/20 ratio. The high-risk and low-risk groups in the test set are identified using the prognostic index (**PI**), which is the linear component of the Cox model: $\mathrm{PI}=\beta^{T}X_{\text{test}}$, where $X_{\text{test}}$ is the omics profile of the test set, and the risk coefficients were estimated from the trained Cox model on the training set. The test set samples were divided into two equally sized groups using the median value of **PI**. The log-rank test *P*-value was calculated to compare the difference between the two groups. We implemented the Cox model using Python’s *scikit-survival* package (Pölsterl 2020), and the *lifelines* package (Davidson-Pilon 2019) was used for the Kaplan–Meier plots.

## 3 Results

### 3.1 Datasets

The experiments on our proposed framework were run on The Cancer Genome Atlas (TCGA) breast cancer (BRCA) dataset (The Cancer Genome Atlas Network 2012). The two omics profiles used here are RNA-seq mRNA expression and miRNA expression, downloaded from the UCSC Xena Hub (Goldman *et al.* 2020). The dataset contains miRNA expression data for 830 patients. The dataset has mRNA expression data for 1218 patients, including the 830 patients present in the miRNA expression data. There are 20 530 genes with log 2(x+1) transformed RSEM normalized expression values in the mRNA data and 2238 miRNAs with log 2(x+1) transformed RPM feature values in the miRNA data. The clinical information for the breast cancer dataset was obtained from cBioPortal (Gao *et al.* 2013). For phenotype prediction, we predict the Estrogen Receptor and Triple Negative (TN) status of the breast cancer patients. There are 332 Estrogen Receptor positive (ER+) and 80 Estrogen Receptor negative (ER−) samples in the clinical information data. For the TN phenotype, the dataset has 65 TN and 347 non-TN samples.

### 3.2 Imputation and cancer subtype prediction

In this section, we discuss how the two-stage experiment is set up. In the first stage, OmicsNMF is trained and the missing samples are imputed. In the second stage, the imputed omics profile is used for cancer subtype prediction to ensure that the imputed data contains meaningful features for downstream tasks.

The experiments were conducted for two cases: first, to impute miRNA from mRNA, and second, to impute mRNA from miRNA. For mRNA, we have 1218 samples, and for miRNA, we have 830 samples. Therefore, when imputing miRNA from mRNA, we have 388 missing samples. We split the 830 miRNA samples into training and validation sets, with 80% being training data. The 388 samples that are missing for miRNA are available in the mRNA data as source omics. However, when imputing mRNA from miRNA, we keep 20% of the available 830 mRNA samples separated as missing samples. These samples are available in the miRNA data as source omics. We then split the remaining samples into training and validation sets with an 80/20 ratio. We performed 100 random splits following these conditions to avoid any data bias. For each of these hundred splits, OmicsNMF was trained. The validation data was used only to evaluate the model’s performance and to monitor convergence. The samples which are available on both source and target will be used to calculate both MSE and NMF loss during training. For NMF decomposition, we used a cluster size of 10 (ie, *c *=* *10). Additionally, as discussed in Section 2.2 the samples that are missing on the target omics but available in the source omics will still be used to calculate the NMF loss and assist the training via the second term in Equation (5). For evaluation we use the validation split to calculate the MSE for the generated or imputed samples.

**Figure 2.**
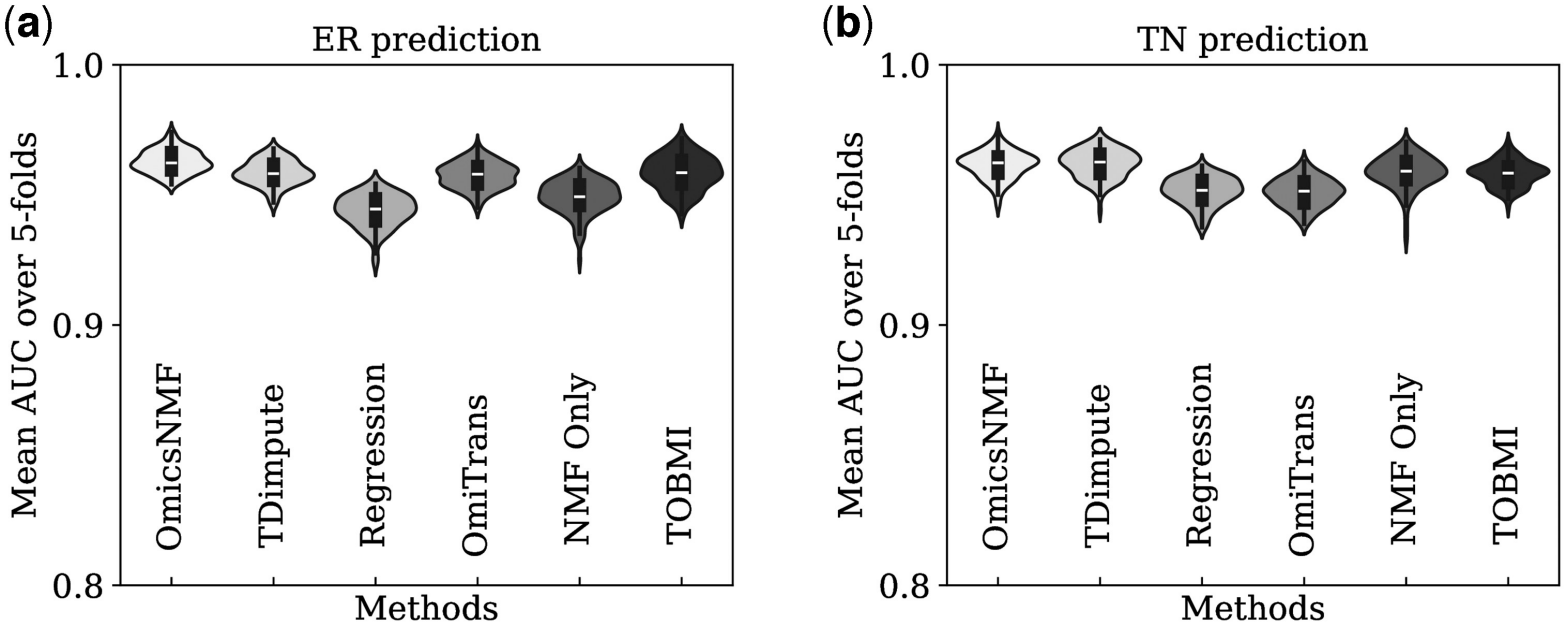
Classification results using imputed microRNA. Average 5-fold validation AUC result distribution over 100 random splits for different methodologies on the mRNA to microRNA imputation task for (a) ER prediction and (b) TN prediction by the random forest classifier.

**Figure 3.**
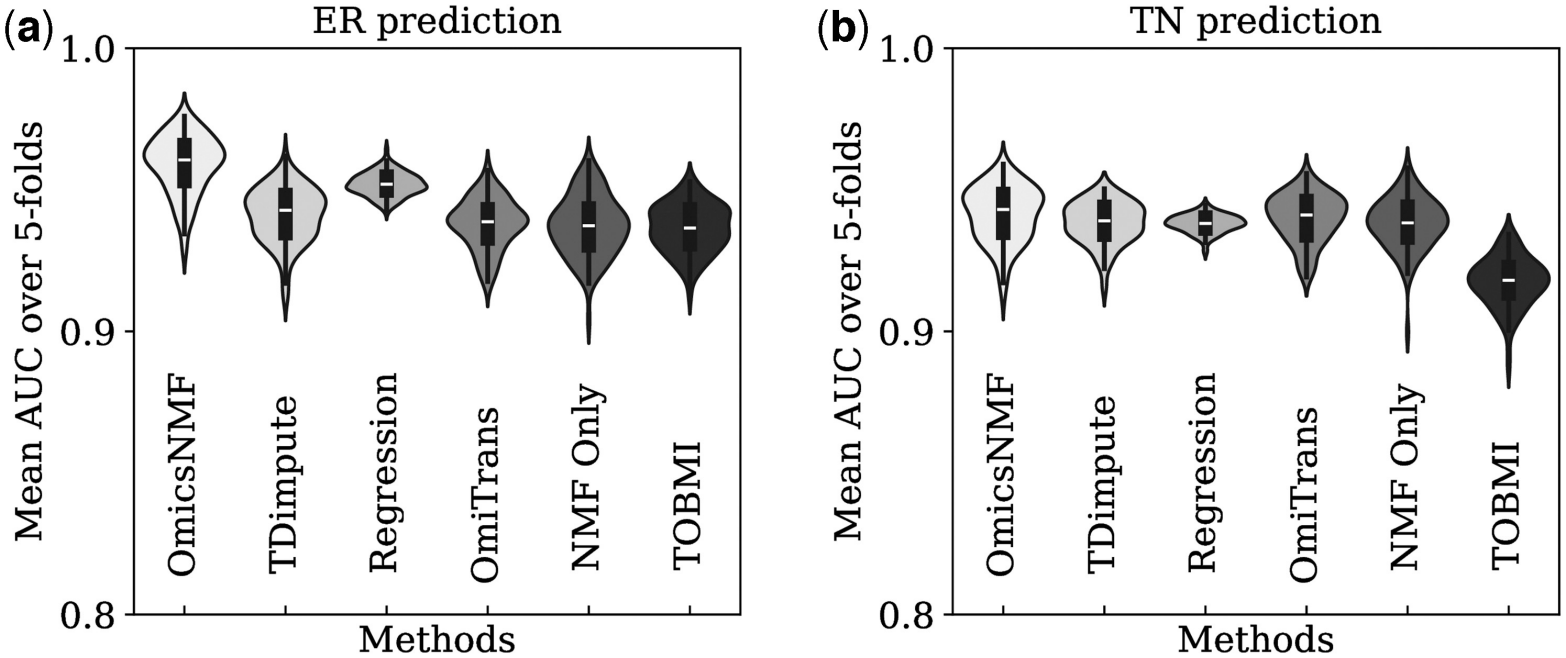
Classification results using imputed mRNA. Average 5-fold validation AUC result distribution over 100 random splits for different methodologies on the microRNA to mRNA imputation task for (a) ER prediction and (b) TN prediction by the random forest classifier.

To evaluate the contribution of the imputed data in cancer subtype prediction, a Random Forest Classifier was used. The imputed training, validation, and missing samples were used for a 5-fold cross-validation on the Random Forest Classifier for the prediction task. The average AUC (Area Under the Curve) was calculated over the 5-fold cross-validation for both ER and TN prediction as the performance metric. Since the GAN was trained using 100 different data splits, the 5-fold cross-validation was performed after training on each split. The AUC results from the 5-fold cross-validation were then averaged over the hundred splits and reported as the *Overall* AUC in Tables 2 and 3. Figures 2 and 3 show the distribution of the average AUC across the 100 different splits for various methods.

**Table 2.** Phenotype classification performance comparison using imputed microRNA.

		OmicsNMF	TDimpute	Regression	OmiTrans	NMF only	TOBMI
Overall	ER	**0.963**	**0.958** ^*^	0.944^*^	0.957^*^	0.949^*^	0.958^*^
	TN	**0.961**	**0.962**	0.951^*^	0.951^*^	0.959^*^	0.958^*^
Test set	ER	**0.974**	0.967^*^	0.923^*^	0.967^*^	**0.970** ^*^	0.969^*^
	TN	**0.981**	**0.981**	0.955^*^	0.981	0.979	0.979

The performance of different methods compared to omicsNMF on the mRNA to microRNA imputation task is presented. The “Overall” AUC represents the average AUC from 5-fold cross-validation over 100 training splits. The AUC on “Test set” shows the AUC by evaluating on the test set only, averaged over the 100 splits. Both AUC values are reported separately for TN and ER prediction. The best two results are bolded. The (${}^{*}$) marks indicate that OmicsNMF is statistically significant than the marked methods with a *P*-value <.05.

**Table 3.** Phenotype classification performance comparison using imputed mRNA.

		OmicsNMF	TDimpute	Regression	OmiTrans	NMF only	TOBMI
Overall	ER	**0.959**	0.941^*^	**0.952** ^*^	0.938^*^	0.937^*^	0.936^*^
	TN	**0.941**	0.938^*^	0.938^*^	**0.940**	0.938^*^	0.917^*^
Test set	ER	0.932	**0.937**	0.936	0.933	0.934	**0.965**
	TN	**0.893**	0.887	**0.898**	0.884	0.883^*^	0.876^*^

The performance of different methods compared to omicsNMF on the microRNA to mRNA imputation task is presented. The “Overall” AUC represents the average AUC from 5-fold cross-validation over 100 training splits. The AUC on “Test set” shows the AUC by evaluating on the test set only, averaged over the 100 splits. Both AUC values are reported separately for TN and ER prediction. The best two results are bolded. The (*) marks indicate that OmicsNMF is statistically significant than the marked methods with a *P*-value <.05.

We also evaluated the quality of the imputed data by training the Random Forest Classifier using the training and validation samples and then testing it on the imputed missing samples. We refer to this as the Test set in Tables 2 and 3, which consists of the samples assumed missing from the initial dataset and imputed after training OmicsNMF. This performance on the test set represents the effectiveness of the downstream task on the completely missing samples after imputation, with the Random Forest Classifier trained only on the available samples. From the Tables 2 and 3 it is evident that OmicsNMF consistently performs better than the other baseline methods. However, in some cases, other methods may perform slightly better, but even in those instances, our method performs on par with them.

The MSE between the available omics and the generated omics was calculated for the samples in the validation split. From Table 4, it is observable that OmicsNMF does not have the lowest MSE loss for the imputation task. However, OmicsNMF performs competitively with other methods, given that most baseline methods directly or indirectly aim to minimize only the MSE loss during the training phase. In contrast, OmicsNMF is a GAN-based method with an advanced objective function that seeks to minimize both the MSE loss and the NMF loss, along with the adversarial loss. Therefore, despite not achieving the lowest MSE loss, our proposed method demonstrates competitive performance by optimizing a multi-faceted training objective, balancing MSE, NMF, and adversarial losses. From the classification results of the “NMF Only” model in Tables 2 and 3, we see that it can impute samples with a high MSE loss but still preserve important features for predicting breast cancer subtypes. However, it is also essential to keep the reconstruction loss as low as possible. The MSE loss helps the GAN model steer the imputation towards better reconstruction error while conserving the features.

**Table 4.** MSE loss comparison between different methods.

Method	mRNA to microRNA	microRNA to mRNA
OmicsNMF	0.692	1.801
TDimput	0.744	1.911
Regression	0.515	1.467
OmiTrans	0.634	1.956
NMF only	29.196	49.699
TOBMI	0.701	1.984

As MSE loss is an important metric for imputation, it can be observed from the tables that the methods with the minimum MSE are not always the best performing in terms of TN or ER classification. This suggests that the NMF loss is conserving meaningful features for the imputed samples, leading to better performance on downstream tasks. Furthermore, the formulation of NMF loss, as discussed in Section 2.2, shows that it enables the use of source omics samples that are completely missing in the target omics for training. This is not possible when training a model using only MSE loss. This advantage of our framework makes it more reliable for practical use, as the imputation is not entirely dependent on the training data and the fitted model. Instead, it incorporates source omics information through the cluster centroid loss for imputation.

### 3.3 Survival analysis

**Figure 4.**
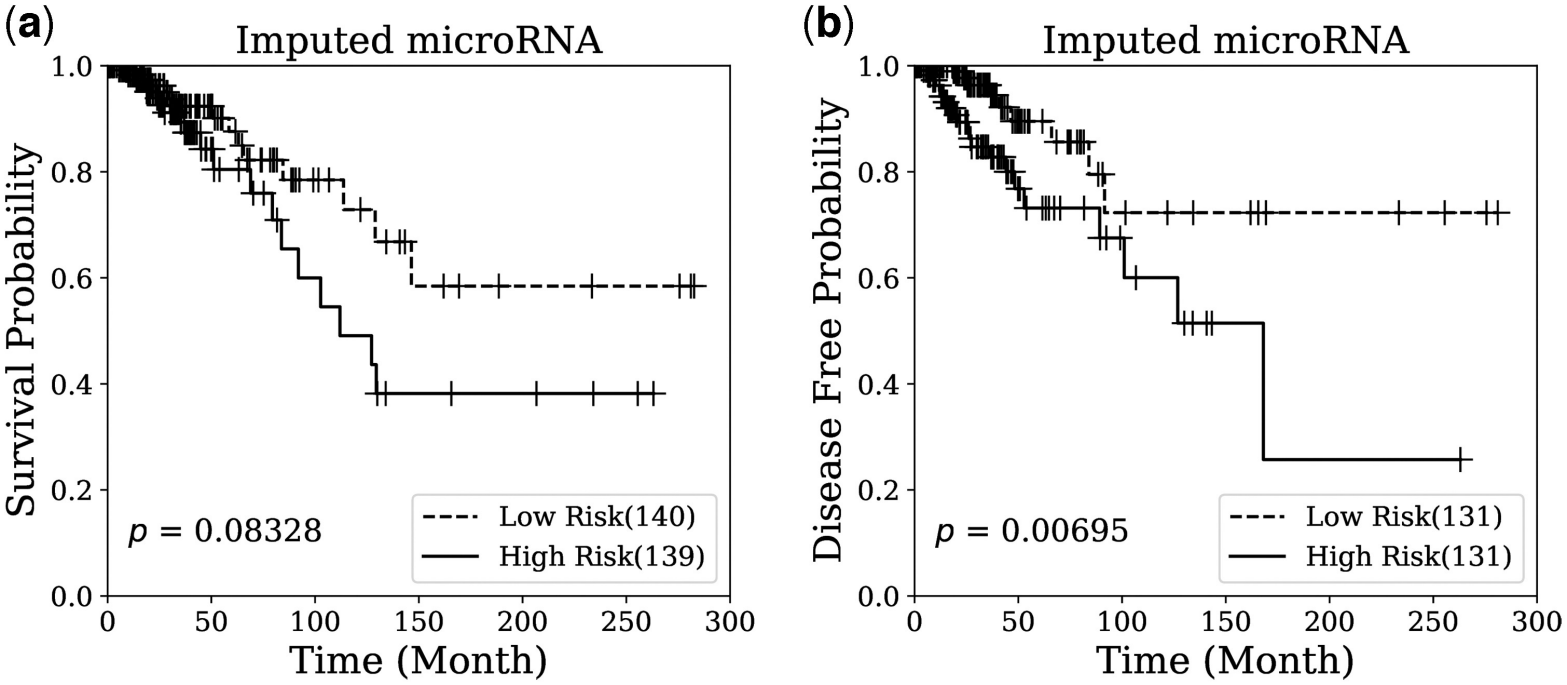
Kaplan–Meier survival analysis on breast cancer patients using imputed microRNA. The imputed features were used for (a) survival prediction and (b) disease free prediction on breast cancer patients. The number of samples in low or high risk group is indicated by the number in the parenthesis. Log-rank test is used to calculate the *P*-value for the comparison between two risk groups.

**Figure 5.**
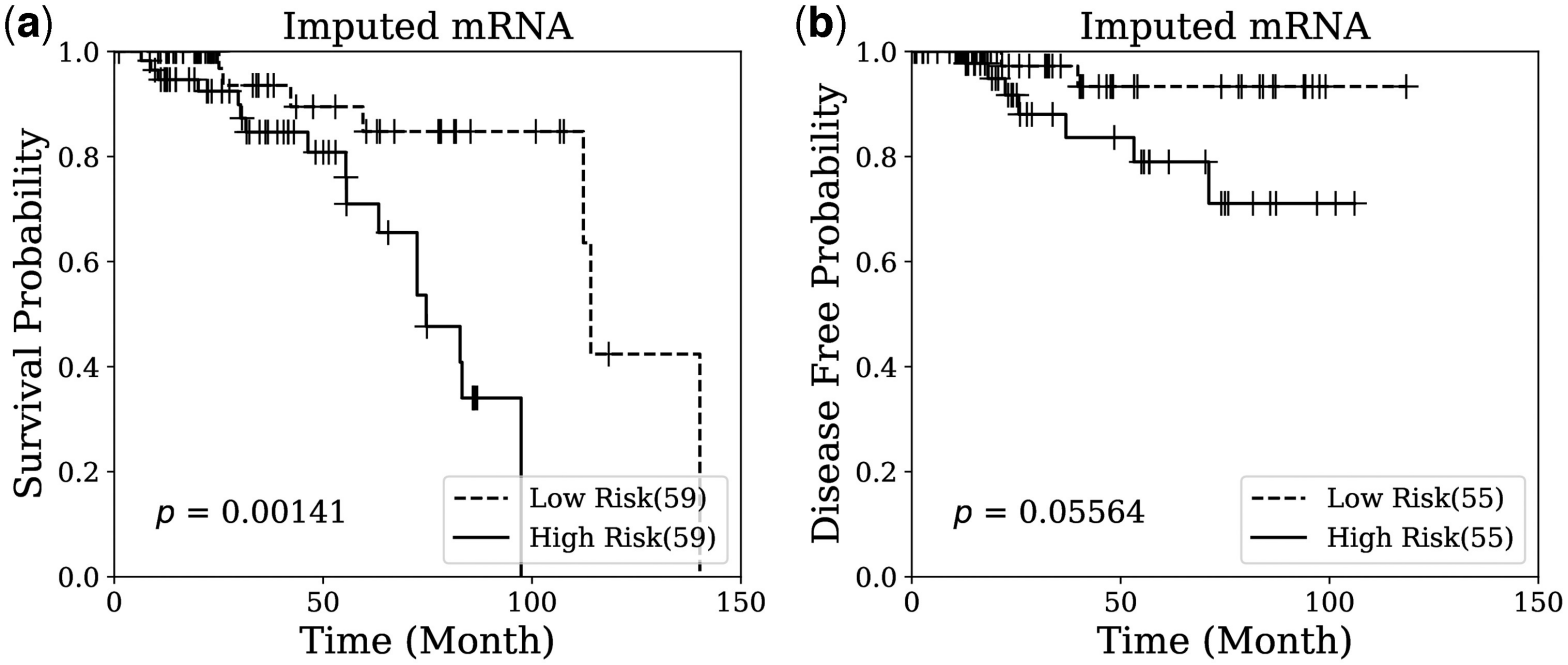
Kaplan–Meier survival analysis on breast cancer patients using imputed mRNA. The imputed features were used for (a) survival prediction and (b) disease free prediction on breast cancer patients. The number of samples in low or high risk group is indicated by the number in the parenthesis. Log-rank test is used to calculate the *P*-value for the comparison between two risk groups.

To ensure the quality of the imputed data, survival analysis was conducted on the breast cancer dataset to predict overall survival and disease-free status. The analysis utilized the Cox proportional hazards model with Elastic Net penalty, detailed in Section 2.3. The dataset was partitioned into training and testing sets using the method outlined in Section 3.2. Following model training, the prognostic index **PI** was computed for the test set, enabling the separation of high-risk and low-risk groups for generating Kaplan–Meier plots. Figures 4 and 5 display the Kaplan–Meier plots generated by microRNA and mRNA, respectively, for the imputed data from the test set using our proposed method. The distinct separation observed between the high-risk and low-risk groups in both survival and disease-free status plots indicates that the imputation quality of OmicsNMF is robust and meaningful. Additionally, the log-rank test *P*-values confirm the significant prognostic power of the imputed omics profiles in survival analysis.

## 4 Discussion

GAN-based models have already proven to be excellent in generative tasks by minimizing adversarial loss. In this study, we formulated the imputation task as a generation problem to harness the power of GANs. However, differentiating between real and imputed omics samples is challenging due to the inherent high dimensionality of omics data. As a result, relying solely on adversarial loss is insufficient for training a GAN for omics data generation, especially with low sample sizes. NMF is well-suited for handling large-scale matrices. While MSE helps maintain the similarity between the generated and original data, NMF can identify the underlying patterns in omics data by decomposing it, thus ensuring the preservation of biological characteristics.

From Table 4, it is observable that our framework does not achieve the lowest MSE loss for the imputed data compared to the baselines as most baselines directly optimize their models by minimizing MSE loss during training. However, OmicsNMF optimization is based on three different loss terms, as shown in Equation (5), resulting in a slightly higher MSE that comes with a significant reward in terms of more meaningful feature generation. As shown in Tables 2 and 3, the methods with the lowest MSE loss are not always the best for breast cancer subtype prediction tasks, suggesting a significant contribution of NMF loss in OmicsNMF for generating features with discriminative power. The inclusion of test data during training through NMF provides limited supervision for the samples that need imputation, rather than relying solely on the model trained on the available data. However, this advantage diminishes when a significant portion of the data is missing and requires imputation. For OmicsNMF to accurately estimate the centroids of the complete dataset post-imputation, a majority of the data must be available. To mitigate this issue, a smaller value of *α* in Equation (5) can be used, which emphasizes the importance of adversarial loss and MSE loss in model training.

OmicsNMF is designed to impute omics profiles from a single source omics dataset. Although our study focused on microRNA and mRNA datasets, this method can be applied to any two types of omics data. Additionally, integrating multiple omics profiles has proven to be more effective for downstream tasks, suggesting that imputation from multiple source omics profiles, rather than a single source, could enhance imputation accuracy. A more complex generator module could be developed to take multiple source omics as input and generate the target omics from the combined information. Future research focused on developing and refining these multi-omics integration methods could fully leverage the potential of diverse biological datasets for complete missing omics sample imputation.

Our experiments were conducted on an NVIDIA RTX A4500 GPU. The framework uses only 1GB of vRAM during execution due to the relatively small dataset size: 1218 patients with 20 530 mRNA features and 2238 microRNA features, respectively. Therefore, the framework can be implemented on a smaller GPU without any increase in runtime. OmicsNMF requires approximately 25 minutes to complete 50 epochs, including the NMF decomposition for this dataset. The pipeline should remain computationally efficient even with larger omics datasets, as long as the generator and critic architectures are not excessively large.

## 5 Conclusion

In this study, we introduced OmicsNMF, a GAN-based model designed to impute missing values in completely missing samples within multi-omics data. The novel objective function in OmicsNMF incorporates NMF loss, aligning the optimization of the model with underlying omics patterns to generate missing values while preserving genomic features. We observed that the imputed dataset has strong discriminative power for cancer outcome prediction, and its consistently better performance across different tasks demonstrates the robustness of the framework. Although OmicsNMF does not always achieve the lowest MSE loss compared to the baselines, it performs very well in downstream tasks. This is because the model is not focused solely on minimizing MSE loss but is optimized through a multi-faceted training objective. Additionally, the NMF loss allows the framework to incorporate all available data, even when some samples are missing in the target omics, enhancing its reliability. Survival analysis using a Cox proportional hazards model indicates the quality of the imputed data, and Kaplan–Meier plots demonstrate the strong prognostic power of the imputed omics profiles for both overall survival and disease-free status in the cancer dataset. In conclusion, OmicsNMF offers a powerful and reliable solution for multi-omics data imputation, providing more accurate and comprehensive analyses in cancer research and other biomedical applications. Future work could explore applying this framework to many-to-one omics imputation tasks and investigate potential enhancements in the GAN architecture to further improve imputation performance.

## Data Availability

The code and datasets of omicsNMF are available at https://github.com/compbiolabucf/OmicsNMF/.

## References

[btae674-B1] Ahmed KT , Sun J, Cheng S et al Multi-omics data integration by generative adversarial network. Bioinformatics 2021;38:179–86. 34415323 10.1093/bioinformatics/btab608 PMC10060730

[btae674-B2] Ahmed KT , Sudipto B, Yanjie F et al Attention-based multi-modal missing value imputation for time series data with high missing rate. In: *Proceedings of the 2023 SIAM International Conference on Data Mining (SDM),* pp. 469–77. Minnesota, USA: SIAM, 2023a.

[btae674-B3] Ahmed TK , Sze C, Qian L et al Incomplete time-series gene expression in integrative study for islet autoimmunity prediction. Brief Bioinform2023b;24:bbac537. 36513375 10.1093/bib/bbac537 PMC9851333

[btae674-B4] Arjovsky M , Soumith C, Leon B. Wasserstein generative adversarial networks. In: *International Conference on Machine Learning*, pp. 214–23. Sydney, Australia: PMLR, 2017.

[btae674-B5] Cho K , van Merrienboer B, Gulcehre C et al Learning phrase representations using RNN encoder-decoder for statistical machine translation. arXiv, 10.48550/arXiv.1406.1078, 2014, preprint: not peer reviewed.

[btae674-B6] Davidson-Pilon C. Lifelines, survival analysis in Python. *JOSS* 2029;**4**:1317.

[btae674-B7] Dong X , Lin L, Zhang R et al TOBMI: trans-omics block missing data imputation using a k-nearest neighbor weighted approach. Bioinformatics 2019;35:1278–83. 30202885 10.1093/bioinformatics/bty796

[btae674-B8] Gao J , Aksoy BA, Dogrusoz U et al Integrative analysis of complex cancer genomics and clinical profiles using the cBioPortal. Sci Signal 2013;6:pl1. 23550210 10.1126/scisignal.2004088 PMC4160307

[btae674-B9] Goldman MJ , Craft B, Hastie M et al Visualizing and interpreting cancer genomics data via the xena platform. Nat Biotechnol 2020;38:675–8. 32444850 10.1038/s41587-020-0546-8 PMC7386072

[btae674-B10] Gomez-Cabrero D , Abugessaisa I, Maier D et al Data integration in the era of omics: current and future challenges. BMC Syst Biol 2014;8 Suppl 2:I1–10. 10.1186/1752-0509-8-S2-I1 PMC4101704 25032990

[btae674-B11] Goodfellow IJ , Pouget-Abadie J, Mirza M et al Generative adversarial nets. In: Advances in Neural Information Processing Systems, Montreal, Canada, 2014, 27.

[btae674-B12] Hawe JS , Theis FJ, Heinig M et al Inferring interaction networks from multi-omics data. Front Genet 2019;10:535. 31249591 10.3389/fgene.2019.00535 PMC6582773

[btae674-B13] Isola P, Jun YZ, Tinghui Z et al Image-to-image translation with conditional adversarial networks. In: *Proceedings of the IEEE Conference on Computer Vision and Pattern Recognition*, Hawaii, USA, pp. 1125–34. 2017.

[btae674-B14] Lee DD , Seung HS. Learning the parts of objects by non-negative matrix factorization. Nature 1999;401:788–91. 10548103 10.1038/44565

[btae674-B15] Lee JY , Styczynski MP. NS-kNN: a modified k-nearest neighbors approach for imputing metabolomics data. Metabolomics 2018;14:153. 30830437 10.1007/s11306-018-1451-8 PMC6532628

[btae674-B16] Pölsterl S. scikit-survival: a library for time-to-event analysis built on top of scikit-learn. J Mach Learn Res 2020;21:1–6.34305477

[btae674-B17] Seber GA , Lee AJ. Linear Regression Analysis. New Jersey, United States: John Wiley & Sons, 2012.

[btae674-B18] Simon N , Friedman J, Hastie T et al Regularization paths for cox’s proportional hazards model via coordinate descent. J Stat Softw 2011;39:1. 10.18637/jss.v039.i05 PMC4824408 27065756

[btae674-B19] Stein-O’Brien GL , Arora R, Culhane AC et al Enter the matrix: factorization uncovers knowledge from omics. Trends Genet 2018;34:790–805. 30143323 10.1016/j.tig.2018.07.003 PMC6309559

[btae674-B20] Subramanian I , Verma S, Kumar S et al Multi-omics data integration, interpretation, and its application. Bioinform Biol Insights 2020;14:1177932219899051. 32076369 10.1177/1177932219899051 PMC7003173

[btae674-B21] The Cancer Genome Atlas Network. Comprehensive molecular portraits of human breast tumours. Nature 2012;490:61–70. 23000897 10.1038/nature11412 PMC3465532

[btae674-B22] Tibshirani R. Regression shrinkage and selection via the lasso. J R Stat Soc Ser B Stat Methodol 1996;58:267–88.

[btae674-B23] Tran L , Liu X, Zhou J et al Missing modalities imputation via cascaded residual autoencoder. In: *Proceedings of the IEEE Conference on Computer Vision and Pattern Recognition*, Hawaii, USA, pp. 1405–14. 2017.

[btae674-B24] Troyanskaya O , Cantor M, Sherlock G et al Missing value estimation methods for DNA microarrays. Bioinformatics 2001;17:520–5. 11395428 10.1093/bioinformatics/17.6.520

[btae674-B25] Voillet V , Besse P, Liaubet L et al Handling missing rows in multi-omics data integration: multiple imputation in multiple factor analysis framework. BMC Bioinformatics 2016;17:402. 27716030 10.1186/s12859-016-1273-5 PMC5048483

[btae674-B26] Wörheide MA , Krumsiek J, Kastenmüller G et al Multi-omics integration in biomedical research–a metabolomics-centric review. Anal Chim Acta 2021;1141:144–62. 33248648 10.1016/j.aca.2020.10.038 PMC7701361

[btae674-B27] Wu Y , Burda Y, Salakhutdinov R et al On the quantitative analysis of decoder-based generative models. arXiv, 10.48550/arXiv.1611.04273, 2016, preprint: not peer reviewed.

[btae674-B28] Xu J , Wang Y, Xu X et al NMF-based approach for missing values imputation of mass spectrometry metabolomics data. Molecules 2021;26:5787. 34641330 10.3390/molecules26195787 PMC8510447

[btae674-B29] Yang Z , Michailidis G. A non-negative matrix factorization method for detecting modules in heterogeneous omics multi-modal data. Bioinformatics 2016;32:1–8. 26377073 10.1093/bioinformatics/btv544 PMC5006236

[btae674-B30] Yoon J, James J, Mihaela S et al Gain: missing data imputation using generative adversarial nets. In: *International Conference on Machine Learning*, pp. 5689–98. Stockholm, Sweden: PMLR, 2018.

[btae674-B31] Zhang X , Guo Y. OmiTrans: generative adversarial networks based omics-to-omics translation framework. In: *2022 IEEE International Conference on Bioinformatics and Biomedicine (BIBM)*, pp. 653–9. Nevada, USA: IEEE, 2022.

[btae674-B32] Zhang Y, Z Gan, K Fan et al Adversarial feature matching for text generation. In: *International Conference on Machine Learning*, pp. 4006–15. Sydney, Australia: PMLR, 2017.

[btae674-B33] Zhou X , Chai H, Zhao H et al Imputing missing RNA-sequencing data from DNA methylation by using a transfer learning–based neural network. Gigascience 2020;9:giaa076. 32649756 10.1093/gigascience/giaa076 PMC7350980

